# Overcoming Mohs Limitations in Treating DFSP: Retrospective Analysis of a Novel Excision Technique

**DOI:** 10.3390/life15071025

**Published:** 2025-06-27

**Authors:** Rami Shoufani, Ariel Berl, Ofir Shir-az, Deborah Kidron, Din Mann, Noam Castel, Avshalom Shalom

**Affiliations:** 1 Department of Plastic Surgery, Meir Medical Center, Kfar Saba 4428163, Israel; ramishoufani47@hotmail.com (R.S.); arielberl23@gmail.com (A.B.); ofir.shiraz@gmail.com (O.S.-a.); dinmannlit@gmail.com (D.M.); noamcastel@gmail.com (N.C.); 2 Faculty of Medical and Health Sciences, Tel Aviv University, Tel Aviv 6997801, Israel; 3 Department of Pathology, Meir Medical Center, Kfar Saba 4428163, Israel; dkidron@clalit.org.il

**Keywords:** dermatofibrosarcoma protuberans, Mohs micrographic surgery, recurrence rate, slow-Mohs

## Abstract

Dermatofibrosarcoma protuberans (DFSP) is a rare, soft-tissue sarcoma characterized by dermal, finger-like projections and high local recurrence rates. Complete surgical excision is the primary treatment goal and Mohs micrographic surgery (MMS) is the accepted approach for achieving disease-free margins. Despite the effectiveness of MMS, it has limitations when treating DFSP, with documented local recurrences. This paper presents our experience and treatment modality for DFSP, using MMS with an additional “safety margin”. This technique seeks to ensure free surgical margins and potentially lower recurrence rates. This is a retrospective analysis of patients treated for DFSP over a 10-year period. All patients underwent MMS, followed by an additional, circumferential excision of 2–5 mm. Twenty-two patients were treated surgically for DFSP from 2014 to 2023. The median age at presentation was 42.5 years. Four patients (18%) had positive disease margins on the additional safety marginal excision, two had negative MMS slides, and the other two were positive for DFSP. The mean follow-up time was 27 months, and no local recurrences were observed during that time. The surgical method presented here includes an additional excision of the surrounding margins following MMS for DFSP. This technique provides a tool to overcome the limitations of MMS in treating this tumor, aiming to reduce local recurrence.

## 1. Introduction

Dermatofibrosarcoma protuberans (DFSP) is a rare, locally progressive soft-tissue sarcoma that originates from dermal fibroblasts [1]. Histologically, DFSP is characterized by uniform spindle cells arranged in storiform patterns [2], resulting in extensive, fingerlike DFSP projections into the dermis and underlying adipose tissue [3]. Despite the tendency for local invasion, DFSP rarely metastasizes [4].

DFSP can arise in various anatomical locations, with the limbs and torso the most common sites and the head and neck the least common [5,6]. Clinically, it presents with a range of symptoms, from asymptomatic skin markings to painful ulcerated lesions [7]. Due to its slow growth and often subtle presentation, the diagnosis of DFSP is frequently delayed, resulting in large, deeply infiltrating lesions that necessitate extensive surgical interventions and resections [8].

Complete surgical excision remains the cornerstone of DFSP treatment [9], as achieving clear margins is critical to preventing local recurrence [2] due to the tumor’s propensity for deep-tissue infiltration. It is difficult to achieve clear DFSP margins due to the tumor’s histological overlap with other benign and malignant lesions [3,10,11].

Historically, wide local excision with 2–4 cm margins has been the standard surgical approach. However, this method has been associated with high local recurrence rates, ranging from 6% to 60% [7,12,13], depending on the tumor’s location and the adequacy of the margins. Wide local excision has been associated with higher morbidity due to the extensive amount of tissue removal required, often leading to larger defects and more-complex reconstructions [14,15,16]. Currently, the preferred approach is Mohs micrographic surgery (MMS) [16], a well-established technique for non-melanoma skin cancers [17]. It provides precise control of surgical margins while conserving normal tissue [5,14,18]. Although Mohs micrographic surgery is associated with the lowest recurrence rates for DFSP [7,11], it has some limitations, as local recurrences have been documented in several recent studies [4,5,12,16,19,20,21]. These recurrences are primarily due to the failure to completely remove the tumor’s vertical and horizontal projections, which underscores the importance of the thorough evaluation of all peripheral and deep margins in DFSP surgery.

The aim of this study was to review and present our experience using a novel technique for the treatment of DFSP. It involves an additional stage to the traditional Mohs micrographic surgery approach in order to overcome the limitations of Mohs micrographic surgery and ensure negative excisional margins, thereby reducing local recurrence rates and minimizing morbidity.

## 2. Materials and Methods

### 2.1. Study Design

This retrospective study included a review of all patents with pathologically confirmed DFSP, who were treated with Mohs micrographic surgery in the Plastic and Reconstructive Surgery Department at Meir Medical Center, Israel, from 2014 to 2023.

### 2.2. Ethics

This study was approved by the Institutional Review Board (approval number: MMC-0096-23).

### 2.3. Study Cohort

Incident cases included all patients with pathologically confirmed DFSP who underwent curative-intent surgical treatment with out-patient Mohs micrographic surgery at our institution. Data were extracted from electronic health records and included demographic and clinical information, lesion location and size, type of initial biopsy, histological tumor composition, method of reconstruction, size of additional margins excised, surgical complications, surgical margins, postoperative functional results, local recurrence or metastasis, imaging studies, and other oncological consultations.

### 2.4. Surgical Technique

Patients diagnosed with DFSP underwent Mohs micrographic surgery according to the standardized excisional technique. First, the suspected lesion or DFSP-confirmed scar was marked for excision with macroscopically free margins. The excised tissue was then marked for orientation, processed using the Mohs micrographic surgery frozen section technique, and analyzed immediately by a certified dermatopathologist. If any residual DFSP was detected, further margin excision was performed in the same manner until disease-free surgical margins were achieved. The next stage was performed only after disease-free surgical margins were achieved using the standard Mohs micrographic surgery technique.

**Figure 1:**
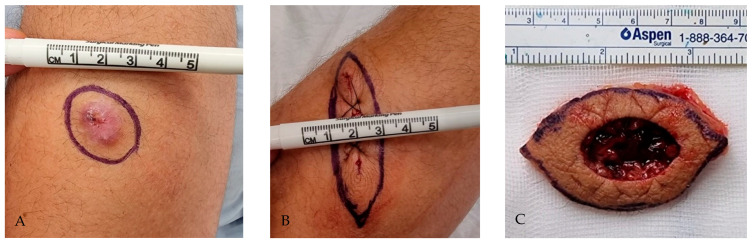
Additional surrounding excisional margin technique. A 34-year-old male presented with DFSP of the right arm. (**A**) DFSP lesion on upper limb; excisional margins are highlighted, with macroscopic DFSP-free margins, and excised using MMS. (**B**) After negative MMS surgical margins were obtained with the frozen section technique, an additional excisional margin was highlighted for excision and paraffin-embedded pathological examination. (**C**) Additional “safety” margin specimen following excision.

The surgical approach presented here includes an additional surgical stage performed after the Mohs micrographic surgery. After confirming residual DFSP-free margins on frozen Mohs micrographic surgery sections, an additional 2–5 mm ring of tissue surrounding the surgical defect, with a similar depth as the Mohs micrographic surgery excision, is removed. The width of the additional excision is determined intraoperatively depending on the location of the lesion. This additional specimen is marked for orientation and sent for paraffin-embedded pathological evaluation of the excisional margins. The technique is further depicted in Figure 1.

### 2.5. Patient Follow-Up

The analysis of pathological specimens includes a review of the Mohs micrographic surgery slides and the additional “safety” excision ring. These are reviewed by a certified dermatopathologist to confirm negative DFSP margins. In cases where the additional ring had positive surgical margins, patients were scheduled for another Mohs micrographic surgery excision using a similar technique to achieve DFSP-free margins. In all cases of a positive result in the “safety” ring, the previous slides were reviewed pathologically to confirm the diagnosis prior to the additional surgery.

All patients were followed-up postoperatively at regular intervals: every 6 weeks for the first 3 months, every 6 months for the next 3 years, and annually thereafter. Clinical and dermoscopic examinations were routinely performed, along with imaging studies including chest CT and ultrasound or MRI of the scar, to rule out recurrence. Follow-up duration was calculated from the date of the initial Mohs micrographic surgery procedure to the last clinical visit.

### 2.6. Data Analysis

Demographic data, skin lesion location and size, presurgical imaging workup, surgical data, early and late complications, and pathology results were recorded using an Excel spreadsheet (Microsoft Corp., Redmond, WA, USA). The study variables were evaluated and analyzed to study distributions and correlations with patient outcomes. Descriptive and univariate statistical analyses were performed using SPSS-21 (IBM Corp., Armonk, NY, USA).

## 3. Results

A total of 25 patients underwent surgical treatment for DFSP during the study period. Three were lost to the follow-up, leaving 22 patients in the cohort, of which 16 (73%) were male. The median age at presentation was 42.5 years (range: 28–72). None of the patients had undergone curative surgery before presenting to our medical center.

Lesion distribution was as follows: 6 (27%) were located on the head and neck, 7 (31%) on extremities, and 3 (14%) each on the chest, abdomen, and back. Patient demographics are shown in Table 1.

Using Mohs micrographic surgery, complete tumor excision (R0) was achieved in all patients, as confirmed by the intraoperative pathology reports. A 2–5 mm ring of tissue was also excised as a safety margin in all cases, as described in the Methods section above.

Analysis of the pathology reports showed that all cases were consistent with classic DFSP. The additional “safety” ring excised was positive for DFSP in four male patients (18%), with an average age of 39.5 years. Three of the four cases were in the scalp region; the fourth case was on a lower extremity. These four patients underwent a second Mohs surgical excision, employing the same technique as described above. The second Mohs micrographic surgery revealed malignant DFSP cells in the excised specimen, necessitating the further excision of the margins. Mohs micrographic surgery was continued until negative margins were achieved, and an additional surrounding “safety” ring was excised. This additional ring was found to be negative for malignancy in all four cases.

**Figure 2:**
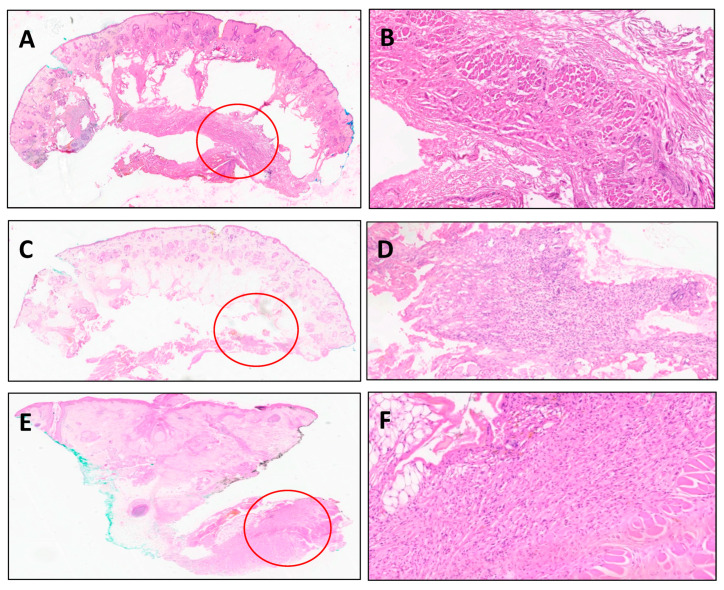
A 32-year-old male who underwent excision of dermatofibrosarcoma protuberans (DFSP) involving the forehead. (**A**,**B**) Frozen section showing no tumor in deep aspect (**A**). Low magnification, H&E X0.5. (**B**). High magnification, H&E X10). (**C**,**D**) Formalin-fixed paraffin-embedded section of the same tissue: a minute nest of DFSP adjacent to a layer of skeletal muscle, underneath subcutaneous adipose tissue. (**C**). Low magnification, H&E X0.5. (**D**). High magnification, H&E X10). (**E**,**F**). An additional focus of DFSP in deep subcutaneous tissue, adjacent to skeletal muscle, in the specimen of the ring-shape wide excision (**E**). Low magnification, H&E X1. (**F**). High magnification, H&E X10).

After analyzing the results, we conducted another review of these four cases and of all the slides, with a certified pathologist. In our review, we found that two of the cases with positive margins on the additional ring had positive DFSP findings on the initial frozen Mohs micrographic surgery slides, which had been deemed negative on intraoperative analysis. Conversely, the other two cases had negative margins on both intraoperative Mohs micrographic surgery results and following our review. The paraffin-embedded “safety” rings were positive for DFSP. Figure 2 illustrates representative histologic images from frozen sections and the formalin-fixed paraffin-embedded section safety ring specimens, in a case where discrepancies occurred.

The mean follow-up duration was 27 months (range: 14–88), and none of the patients presented with local recurrence. All patients were referred for an annual oncological evaluation (in addition to our follow-up), and none required neoadjuvant radiation therapy. Patients underwent annual systemic radiological screening, with no evidence of secondary metastasis. Additionally, 10 of the 22 patients underwent ultrasonographic imaging of the lymph node basin associated with the lesion, and all results were negative.

## 4. Discussion

DFSP is a rare, low-grade sarcoma with a notorious tendency for local recurrence [2]. The primary treatment modalities of Mohs micrographic surgery and wide local excision [5] aim to achieve complete surgical excision. Several series, including a review by Farma et al. [9], have shown that Mohs micrographic surgery provides a lower risk of inadequate margins [5,14,18], and thus is associated with recurrence rates as low as 2–3% [1,9,14,19,20,21,22] and up to 6% [23] in some reports. Furthermore, the use of Mohs micrographic surgery allows the surgeon to preserve uninvolved tissue, which is especially important in the face or other functional areas.

Various risk factors contribute to local recurrence following DFSP resections, which can be broadly categorized into two groups: tumor-related factors and procedural factors.

### 4.1. DFSP Tumor-Related Factors

Tumor-related factors encompass the biological and histopathological characteristics intrinsic to DFSP, which significantly impact surgical outcomes. These factors include finger-like projections radiating into the surrounding normal tissues [3,12], tumor size [19], increased cellularity, increased mitotic rate [8,24], subtype, such as fibrosarcomatous transformation [25], and location of the lesion [9,23]. In the current study, three of the four cases with positive margins on the additionally excised ring were located on the scalp, a region associated with a higher risk of local recurrence, reaching 43% in some studies [6,9,20,23,24] as it has a locally more aggressive nature [7,20].

### 4.2. Procedural Factors

The second group of risk factors is associated with surgical technique and pathological processing and evaluation. These include difficulties in accurately assessing margins intraoperatively, as the distance to DFSP-free surgical margins remains the critical prognostic factor in reducing recurrence [24,26]. Procedural factors also encompass potential errors in tissue handling [27], such as issues with preparing frozen sections [23] and difficulties with pathological analysis, as DFSP foci can mimic normal tissue histologically [19]. Additionally, when preparing the frozen section slides, areas of subcutaneous fat can smear or crumble during sectioning, which in turn can lead to poor-quality slides and incomplete margin assessment. This affects the pathologist’s ability to properly assess DFSP foci when they are located in the deep fat.

Other factors include tumor cell seeding during surgery [16], fibroblastic proliferation from previous surgery [28], and insufficient pathological evaluation. As highlighted by Toby et al. [29], differentiating the deep and lateral margins in a Mohs micrographic surgery slide can be particularly challenging due to the absence of a well-defined plane on microscopy.

Over the course of a decade of experience in treating DFSP, we developed a technique that addresses the challenges of achieving clear DFSP margins. We hypothesized that traditional frozen sections as seen on Mohs micrographic surgery alone might not be sufficient to ensure tumor-free excision, as it has limitations. Our approach involves excising an additional 2–5 mm ring after achieving clear margins on frozen sections during the initial Mohs micrographic surgery. In our experience, this step has proved to be crucial, as 18% of our patients had positive DFSP margins in the additional ring, despite initially negative Mohs surgery results. The use of our technique prevented the misdiagnosis of four patients as R0, with a known risk for recurrence if we had only used previously accepted excision protocols.

In order to learn more about these findings, we reexamined the initial pathology slides of the cases with the positive ring with a certified dermatopathologist. Negative frozen section Mohs micrographic surgery slides were observed in half the cases; however, in the other 50%, the frozen section slides showed positive DFSP cells, with the hypercellularity characteristic of DFSP.

Categorizing risk factors into tumor-related and procedural factors aligns closely with our results. Our findings suggest that the discrepancy between negative Mohs micrographic surgery slides and positive margins on the additional excisional ring may be attributed to the tumor’s aggressive and infiltrative growth patterns, which often extend beyond what is detectable during standard intraoperative frozen section evaluations. This highlights the inherent challenges of achieving clear margins despite surgical precision. The 50% of cases with positive margins could be linked to surgical and pathological factors, underscoring the limitations of using Mohs micrographic surgery exclusively in treating DFSP, where multiple factors can affect the results of the margins.

Several studies reported their experience with wide-excision surgeries resulting in low recurrence rates (4–8%) after an average follow-up of 50 months [15]. However, up to 50% of these cases required extensive reconstruction, with recurrence rates reaching 6.8% [11]. The use of wide excisions to prevent local recurrence might seem appealing. Yet, this technique has several flaws when compared to Mohs micrographic surgery. These include but are not limited to intraoperative evaluation of surgical margins and the removal of uninvolved tissue, which may require larger and more-complex reconstructions [11]. Furthermore, as shown in previous studies, the use of a wide excision can still lead to local recurrence rates that are higher than if Mohs micrographic surgery was employed [1,9,11,14,19,20,21,22,23].

Aiming to definitively achieve complete resection in excised tumors, many surgeons have begun to use slow-Mohs micrographic surgery [21,30,31], which employs paraffin-embedded sections and horizontal slices for more-precise margin evaluation. Slow-Mohs surgery tissue handling eliminates a drawback of frozen Mohs surgery, with less tissue tearing and fat loss while preparing the frozen sections [21,32]. Chaput et al. [33] reported good outcomes with paraffin Mohs micrographic surgery. Lee et al. [21] compared the outcomes of frozen versus paraffin Mohs micrographic surgery when operating on DFSP cases and reported similarly effective recurrence rates.

Although slow-Mohs micrographic surgery could minimize the procedural risk factors, it does not address the tumor-related factors. Furthermore, these papers stated that frozen Mohs micrographic surgery had the advantage of shorter surgery time and immediate closure [21], with lower morbidity. Another inherent limitation of using the slow-Mohs technique is that the surgical defect must be left open until the pathology report is received, which can take several days. Leaving an open wound has its drawbacks, including patient discomfort, tissue desiccation, and risk of infection. Our technique uses traditional MMS with an additional safety margin, eliminating the need to leave an open wound until the pathology is completed. This approach was shown to be effective, as the additional safety margin prevented the misdiagnosis of cases with positive margins, which would have been considered R0 if relying on the initial Mohs micrographic surgery alone.

To date, the follow-up of the patients who underwent DFSP removal using our described technique has not yielded any local recurrences. The lack of recurrences highlights the efficacy of our technique compared with other studies [3,5,9,12,18,20,22,26,32]. This outcome is particularly notable given that the median time to recurrence reported in the literature is approximately 32 months [24]. Our findings underscore the importance of long-term clinical follow-up, especially during the first three years post-surgery, when most recurrences are likely to occur [1,22,24].

The goal of Mohs micrographic surgery is to achieve fully negative margins while preserving as much uninvolved tissue as possible; however, our findings, along with data from multiple studies, indicate that local recurrence can still occur. This highlights the limitations of Mohs micrographic surgery in treating DFSP and emphasizes the critical need for an additional step or modification to the standard Mohs micrographic surgery technique to further reduce the risk of recurrence.

The present study is the first to thoroughly test the efficacy of this modified technique, although similar approaches have been suggested. Tom et al. [34] reported using an inverted horizontal paraffin sectioning method, which revealed residual tumors in seven of nine cases in the head and neck region, highlighting the limitations of margin evaluation with Mohs micrographic surgery alone. Similarly, Tan et al. [35] proposed a 3 mm margin excision during Mohs surgery for DFSP but did not provide details on the pathological examination of the additional tissue. Other studies [36,37] have recommended excising an additional margin ring to address challenges in distinguishing DFSP spindle cells from fibroblasts or scar tissue. However, these techniques were neither fully detailed nor systematically evaluated. Our findings substantiate the value of our technique of the supplementary excision of an additional ring of tissue, demonstrating its potential to improve the reliability of achieving tumor-free margins and decreasing local recurrence rates in the management of DFSP.

As with all studies, this one had several limitations. It was a single-center study, and all procedures were performed using the same protocol, which may limit the generalizability of the findings. Also, none of the patients required neoadjuvant treatment, potentially reflecting a cohort with more moderate disease, with a lower risk of recurrence. Additionally, the average follow-up period in our outpatient clinic was 27 months. This relatively limited duration compared to previous studies is because most patients are still in the early stages of the follow-up, which is shorter than the average risk interval for local recurrence [24]. Finally, the pathology analysis did not include the subtype of DFSP, despite advances in genomics that could prove insights into recurrence risk based on tumor subtype [2].

## 5. Conclusions

The technique for treating DFSP, which involves the removal of an additional ring of tissue after achieving clear margins with Mohs micrographic surgery, has been proven to be safe oncologically and can prevent the need for complex reconstructions. This technique has demonstrated effectiveness in overcoming a limitation of Mohs micrographic surgery, ensuring DFSP-free surgical margins with lower recurrences, to date. We believe that this technique is straightforward and effective in improving the attainment of clear margins when treating DFSP. Further research and extended follow-up are warranted to fully evaluate its oncological safety and long-term efficacy.

## Figures and Tables

**Table 1:** Patient demographics and lesion data.

Variable	N (%)
Total patients	22
Male to female ratio	16 (73%):6 (27%)
Median age, y, (range)	42.5 (28–72)
Smoking	0
**Location of Lesion**	
Head and neck	6 (27%)
Chest	3 (14%)
Abdomen	3 (14%)
Back	3 (14%)
Extremities	7 (31%)

## Data Availability

Data will be made available upon reasonable request to the corresponding author.

## Abbreviations

The following abbreviations are used in this manuscript:

DFSP	Dermatofibrosarcoma protuberans
